# Novel triazoles of 3-acetylbetulin and betulone as anticancer agents

**DOI:** 10.1007/s00044-018-2213-x

**Published:** 2018-07-17

**Authors:** Ewa Bębenek, Monika Kadela-Tomanek, Elwira Chrobak, Małgorzata Latocha, Stanisław Boryczka

**Affiliations:** 1 Department of Organic Chemistry, School of Pharmacy with the Division of Laboratory Medicine in Sosnowiec, Medical University of Silesia in Katowice, 4 Jagiellońska Str., 41-200 Sosnowiec, Poland; 2 Department of Cell Biology, School of Pharmacy with the Division of Laboratory Medicine in Sosnowiec, Medical University of Silesia in Katowice, 8 Jedności Str., 41-200 Sosnowiec, Poland

**Keywords:** Betulin, 1,2,3-Triazole, CuAAC, Anticancer activity, Lipophilicity

## Abstract

The CuAAC reaction of azides and acetylenic triterpenes was used for synthesis of new triazoles of 3-acetylbetulin and betulone. The triazole derivatives were evaluated for their anticancer activity in vitro against amelanotic melanoma C-32, ductal carcinoma T47D and glioblastoma SNB-19 cell lines. 28-[1-(3’-Deoxythymidine-5’-yl)-1*H*-1,2,3-triazol-4-yl]carbonylbetulone **6e** exhibited a significant IC_50_ value (0.17 µM) against the human glioblastoma SNB-19 cell line, an almost 5-fold higher potency while compared with reference cisplatin.

## Introduction

The cycloaddition reaction plays an important role in the synthesis of five-membered heterocyclic structures such as 1,2,3-triazoles. Molecules containing a 1,4-disubstituted 1,2,3-triazole ring are prepared regioselectively from azides and terminal alkynes in the copper-(I)-catalyzed azide-alkyne cycloaddition reaction CuAAC (Wei et al. 2012; Marciniec et al. 2017). CuAAC reactions, described by Sharpless and Meldal groups, give high yields under mild conditions and have been used to obtain drugs, photo stabilizers and dyes. Additionally, this reaction occurs in various organic solvents and in aqueous media, in a wide pH area. In contrast to the CuACC reaction, the ruthenium catalyst azide-alkyne cycloaddition is used in the synthesis of the 1,5-disubstituted triazoles (Rostovtsev et al. 2002; Torne et al. 2002; Bonacorso et al. 2013; Bräse et al. 2008; Totobenazara et al. 2015).

Compounds containing 1,2,3-triazole units exhibit interesting biological activities (antimicrobial, anti-inflammatory, anti-tubercular, and antiviral), which has found numerous applications in bioconjugate chemistry and material science. Additionally, 1,4-disubstitued 1,2,3-triazoles show a significant anticancer activity against human cancer cell lines, which are multidrug-resistant (Wang et al. 2010; Dheer et al. 2017; He et al. 2014).

In the last decades, application of 1,3-dipolar cycloaddiction of naturally occurring triterpenes acquired meaning. Conjugation on azides with various alkyne derivatives of pentacyclic triterpenes is designed for the purposes of introduction of the physiologically stable 1,2,3-triazole group (Spivak et al. 2016; Suman et al. 2016; Yu et al. 2013). Most of the triazole analogs of natural compounds have been investigated for their anticancer activity. Majeed et al. synthesized and tested a series of C-3 aryl-substituted 1,2,3-triazoles of betulinic acid for their cytotoxic activity against various human cancer lines like leukemia (HL-60, THP-1), prostate (DU-145, PC-3), lung (A-549), breast (MCF-7), liver (HEP-2), colon (HCT-15), and neuroblastoma (SF-295). The compounds bearing 2-cyanophenyl and 5-hydroxy-1-naphthyl substituted triazole ring exhibited promising IC_50_ values against HL-60 cell line of 2.5 and 3.5 µM, respectively, in comparison to betulinic acid (IC_50_ = 17 µM) (Majeed et al. 2013). In the case of C-28 aryl-substituted 1,2,3-triazoles of betulinic acid, it was observed that compounds containing a 4-fluorophenyl substituted triazole ring had the cytotoxic profile similar to that of betulinic acid. This novel triazole hybrid showed a significant antiproliferative activity in HL-60 (leukemia), MIAPACa2 (pancreas), PC-3 (prostate), and A-549 (lung) cell lines, with IC_50_ values in the range of 5.0–7.0 µM (Khan et al. 2016).

Previously, we described a synthetic route and evaluation of cytotoxicity of betulin and betulone derivatives with a propynoyl group at the C-28 position (Boryczka et al. 2013; Bębenek et al. 2016). Expanding our interest to propynoyl-substituted triterpenes, we converted those acetylenic derivatives into the corresponding 1,2,3-triazoles. In this work, we presented application of the CuAAC reaction in the synthesis of new triazoles of pentacyclic triterpenes and their anticancer activity, as well as lipophilicity properties.

## Material and methods

### General

All organic solvents (from Sigma-Aldrich and P.P.H. STANLAB) were dried and used after purification. Melting points (m.p.) were determined in open capillary tubes on an Electrothermal IA 9300 melting point apparatus and are uncorrected. The ^1^H NMR and ^13^C NMR spectra were recorded on a Bruker Avance III 600 spectrometer in deuterated-d_6_ chloroform (CDCl_3_) or deuterated-d_6_ dimethyl sulfoxide (DMSO) solution. The chemical shifts were reported in ppm (δ), and coupling constant (*J*) values—in hertz (Hz). The spin multiplicity was designated as the singlet (s), doublet (d), triplet (t), quartet (q), and multiplet (m). High-resolution mass spectra (HR-MS) were recorded on a Bruker Impact II instrument. Infrared spectra (IR) were recorded on a Shimadzu IRAffinity-1 FTIR spectrophotometer (Shimadzu, Japan) using the KBr pellet method. The progress of the reactions was monitored by thin layer chromatography (TLC) using silica gel 60 254 F plates (Merck, Darmstadt, Germany) and detected by spraying with a solution of 5% sulfuric (VI) acid and heating to 120 °C. Purity of the obtained compounds was confirmed by column chromatography carried out on silica gel 60, <63 μm (Merck). A mixture of CHCl_3_–EtOH (40:1, 15:1, 5:1 v/v) or CH_2_Cl_2_–EtOH (60:1, 40:1, v/v) was used as the mobile phase.

### Chemistry

#### Synthesis of 3-acetyl-28-propynoylbetulin **3** and 28-propynoylbetulone **4**

3-Acetyl-28-propynoylbetulin **3** was prepared according to the procedure described by Boryczka et al. (Boryczka et al. 2013).

To an ice-cooled (−10 °C) mixture of 3-acetylbetulin **2** (0.48 g, 1 mmol) and propynoic acid (0.12 g, 1.10 mmol) in dichloromethane (5 mL), a freshly prepared solution of dicyclohexylcarbodiimide (0.23 g, 1.12 mmol) and 4-dimethylaminopyridine (0.01 g, 0.08 mmol) in dichloromethane (1 mL) was added. The mixture was allowed to react under argon atmosphere at −10 °C for 5 h. After warming to room temperature, the mixture was stirred overnight. The reaction was monitored by TLC until completion. The resulting precipitate was filtered and the solvent was removed under reduced pressure. The crude product was purified by silica gel column chromatography (CHCl_3_–EtOH 40:1, v/v).

##### 3-Acetyl-28-propynoylbetulin (**3**)

Yield 79%; mp 115–118 °C; R_f_ 0.44 (CHCl_3_–EtOH, 40:1, v/v); IR (KBr) *ν*_max_ 3304, 2946, 2120, 1719, 1457, 1246 cm^−1^; ^1^H NMR (600 MHz, CDCl_3_): *δ* 0.81 (3H, s, CH_3_), 0.86 (3H, s, CH_3_), 0.87 (3H, s, CH_3_), 0.99 (3H, s, CH_3_), 1.05 (3H, s, CH_3_), 2.07 (3H, s, COCH_3_), 2.45 (1H, m, H-19), 2.91 (1H, s, C≡CH), 4.01 (1H, d, *J* *=* 10.8 Hz, H-28), 4.41 (1H, d, *J* *=* 10.8 Hz, H-28), 4.48 (1H, m, H-3), 4.62 (1H, s, H-29), 4.71 (1H, s, H-29); ^13^C NMR (150 MHz, CDCl_3_): *δ* 14.7, 16.0, 16.2, 16.5, 18.2, 19.1, 20.8, 21.3, 23.7, 25.1, 27.0, 27.9, 29.5, 29.6, 34.1, 34.4, 37.1, 37.7, 37.8, 38.4, 40.9, 42.7, 46.4, 47.7, 48.8, 50.3, 55.4, 64.9, 74.6, 74.8, 80.9, 110.0, 149.9, 153.3, 171.0; HRAPCIMS *m/z*: 536.3878 C_35_H_52_O_4_ (calcd. 536.3865).

28-Propynoylbetulone **4** was obtained according to the procedure described by Bębenek et al. The spectra data of acetylenic ester **4** were consistent with those published in the literature (Bębenek et al. 2016).

#### General procedure for the synthesis of triazoles **5a**–**i** and **6a**–**j**

Based on the previously reported method, the acetylenic esters **3**–**4** were converted into triazoles **5a-i** and **6a-j** by reactions with organic azides in toluene in the presence of copper(I) iodide (Bębenek et al. 2017). The copper(I) iodide (0.1 eqv, 0.004 g, 0.02 mmol) and the organic azide (1.05 eqv, 0.21 mmol) were added to a solution of propynoylated derivatives **3** or **4** (0.20 mmol) in toluene (4 mL). Next, the reaction mixture was stirred for another 72 h under reflux. The solvent was evaporated. The crude residue was purified by silica gel column chromatography using various mixtures of organic solvents. The same mobile phases were applied for TLC and in column chromatography (Table 1).

**Table 1:** The mobile phases used in column chromatography and calculated values of the retention factor

Compound	Mobile phase	Ratio	Retention factor R_f_
**5a**	CH_2_Cl_2_–EtOH	60:1	0.39
**5b**	CH_2_Cl_2_–EtOH	40:1	0.55
**5c**	CH_2_Cl_2_–EtOH	60:1	0.32
**5d**	CH_2_Cl_2_–EtOH	60:1	0.43
**5e**	CHCl_3_–EtOH	5:1	0.68
**5f**	CHCl_3_–EtOH	5:1	0.18
**5g**	CH_2_Cl_2_–EtOH	40:1	0.43
**5h**	CHCl_3_–EtOH	15:1	0.45
**5i**	CHCl_3_–EtOH	5:1	0.73
**6a**	CH_2_Cl_2_–EtOH	40:1	0.43
**6b**	CH_2_Cl_2_–EtOH	40:1	0.49
**6c**	CH_2_Cl_2_–EtOH	60:1	0.36
**6d**	CH_2_Cl_2_–EtOH	60:1	0.37
**6e**	CHCl_3_–EtOH	15:1	0.24
**6f**	CHCl_3_–EtOH	5:1	0.22
**6g**	CH_2_Cl_2_–EtOH	60:1	0.31
**6h**	CHCl_3_–EtOH	15:1	0.32
**6i**	CHCl_3_–EtOH	5:1	0.74
**6j**	CHCl_3_–EtOH	5:1	0.20

##### 3-Acetyl-28-(1-benzyl-1*H*-1,2,3-triazol-4-yl)carbonylbetulin (**5a**)

Yield 73%; m.p. 109–111 °C; IR (KBr) *ν*_max_ 3134, 2947, 1732, 1527, 1456, 1246–1193 cm^−1^; ^1^H NMR (600 MHz, CDCl_3_): *δ* 0.85 (3H, s, CH_3_), 0.86 (3H, s, CH_3_), 0.87 (3H, s, CH_3_), 0.99 (3H, s, CH_3_), 1.06 (3H, s, CH_3_), 1.71 (3H, s, CH_3_), 2.06 (3H, s, COCH_3_), 2.51 (1H, m, H-19), 4.13 (1H, d, *J* = 10.8 Hz, H-28), 4.49 (1H, m, H-3), 4.55 (1H, d, *J* = 10.8 Hz, H-28), 4.62 (1H, s, H-29), 4.72 (1H, s, H-29), 5.60 (2H, s, CH_2_), 7.31–7.33 (2H, m, H_Ar_), 7.41–7.44 (3H, m, H_Ar_), 7.97 (1H, s, CH-triazole); ^13^C NMR (150 MHz, CDCl_3_): *δ* 14.2, 14.7, 16.0, 16.2, 16.5, 18.2, 19.1, 20.8, 21.1, 21.3, 23.7, 25.2, 27.1, 27.9, 29.6, 29.8, 34.1, 34.7, 37.1, 37.7, 38.4, 40.9, 42.7, 46.7, 47.7, 48.9, 50.3, 54.5, 55.4, 60.4, 63.6, 80.9, 109.9, 127.1, 128.2, 129.2, 129.3, 133.8, 140.6, 150.1, 161.2, 171.1; HRAPCIMS *m/z* (neg): 668.4474 C_42_H_58_N_3_O_4_ (calcd. 668.4427).

##### 3-Acetyl-28-[1-(4-fluorobenzyl)-1*H*-1,2,3-triazol-4-yl]carbonylbetulin (**5b**)

Yield 63%; m.p. 113–116 °C; IR (KBr) *ν*_max_ 3138, 2963, 1734, 1539, 1457, 1226–1193, 802 cm^−1^; ^1^H NMR (600 MHz, CDCl_3_): *δ* 0.85 (3H, s, CH_3_), 0.86 (3H, s, CH_3_), 0.87 (3H, s, CH_3_), 1.00 (3H, s, CH_3_), 1.06 (3H, s, CH_3_), 1.69 (3H, s, CH_3_), 2.07 (3H, s, COCH_3_), 2.52 (1H, m, H-19), 4.14 (1H, d, *J* = 10.8 Hz, H-28), 4.49 (1H, m, H-3), 4.57 (1H, d, *J* = 10.8 Hz, H-28), 4.62 (1H, s, H-29), 4.72 (1H, s, H-29), 5.57 (2H, s, CH_2_), 7.10–7.13 (2H, m, H_Ar_), 7.31–7.33 (2H, m, H_Ar_), 7.97 (1H, s, CH-triazole); ^13^C NMR (150 MHz, CDCl_3_): *δ* 13.7, 15.1, 15.2, 15.5, 17.1, 18.1, 19.8, 20.3, 22.7, 24.1, 26.1, 26.9, 28.6, 28.8, 33.1, 33.7, 36.0, 36.6, 36.8, 37.4, 39.9, 41.7, 45.6, 46.7, 47.8, 49.3, 52.7, 54.4, 62.6, 79.9, 108.9, 115.3, 115.4, 125.9, 129.1, 129.2, 139.7, 149.0, 160.0, 161.2, 170.0; HRAPCIMS *m/z* (neg): 686.4357 C_42_H_57_FN_3_O_4_ (calcd. 686.4333).

##### 3-Acetyl-28-[1-(4-cyanobenzyl)-1*H*-1,2,3-triazol-4-yl]carbonylbetulin (**5c**)

Yield 56%; m.p. 137–140 °C; IR (KBr) *ν*_max_ 3144, 2949, 2231, 1734, 1540, 1457, 1248–1192 cm^−1^; ^1^H NMR (600 MHz, CDCl_3_): *δ* 0.83 (3H, s, CH_3_), 0.86 (3H, s, CH_3_), 0.87 (3H, s, CH_3_), 1.00 (3H, s, CH_3_), 1.07 (3H, s, CH_3_), 1.70 (3H, s, CH_3_), 2.07 (3H, s, COCH_3_), 2.52 (1H, m, H-19), 4.16 (1H, d, *J* = 10.8 Hz, H-28), 4.49 (1H, m, H-3), 4.58 (1H, d, *J* = 10.8 Hz, H-28), 4.63 (1H, s, H-29), 4.72 (1H, s, H-29), 5.68 (2H, s, CH_2_), 7.40 (2H, d, *J* = 8.4 Hz, H_Ar_), 7.72 (2H, d, *J* = 8.4 Hz, H_Ar_), 8.04 (1H, s, CH-triazole); ^13^C NMR (150 MHz, CDCl_3_): *δ* 14.8, 16.0, 16.2, 16.5, 18.2, 19.1, 19.3, 20.8, 21.3, 23.7, 25.2, 27.1, 27.9, 29.6, 29.8, 34.1, 34.7, 37.1, 37.7, 37.8, 38.4, 40.9, 42.7, 46.7, 47.7, 48.9, 50.3, 53.7, 55.4, 63.8, 80.9, 110.3, 113.3, 117.9, 124.4, 127.3, 128.5, 133.1, 138.9, 141.0, 150.0, 160.9, 171.0; HRAPCIMS *m/z* (neg): 693.4352 C_43_H_57_N_4_O_4_ (calcd. 693.4380).

##### 3-Acetyl-28-(1-phenylthiomethyl-1*H*-1,2,3-triazol-4-yl)carbonylbetulin (**5d**)

Yield 60%; m.p. 105–107 °C; IR (KBr) *ν*_max_ 2945, 1734, 1539,1456, 1247–1194 cm^−1^; ^1^H NMR (600 MHz, CDCl_3_): *δ* 0.86 (3H, s, CH_3_), 0.87 (3H, s, CH_3_), 0.88 (3H, s, CH_3_), 1.01 (3H, s, CH_3_), 1.09 (3H, s, CH_3_), 1.71 (3H, s, CH_3_), 2.07 (3H, s, COCH_3_), 2.51 (1H, m, H-19), 4.14 (1H, d, *J* = 10.8 Hz, H-28), 4.49 (1H, m, H-3), 4.55 (1H, d, *J* = 10.8 Hz, H-28), 4.63 (1H, s, H-29), 4.73 (1H, s, H-29), 5.68 (2H, s, CH_2_), 7.35–7.36 (5H, m, H_Ar_), 8.04 (1H, s, CH-triazole); ^13^C NMR (150 MHz, CDCl_3_): *δ* 14.8, 16.1, 16.2, 16.5, 18.2, 19.1, 20.8, 21.3, 23.7, 25.2, 25.6, 27.1, 27.9, 29.6, 29.8, 34.1, 34.7, 37.1, 37.7, 37.8, 38.4, 40.9, 42.7, 46.7, 47.8, 48.9, 50.3, 54.3, 55.4, 63.6, 68.0, 80.9, 110.0, 126.8, 129.1, 129.7, 131.3, 132.4, 140.6, 150.0, 160.9, 171.0; HRAPCIMS *m/z* (neg): 700.4141 C_42_H_58_N_3_O_4_S (calcd. 700.4148).

##### 3-Acetyl-28-[1-(3’-deoxythymidine-5’-yl)-1*H*-1,2,3-triazol-4-yl]carbonylbetulin (**5e**)

Yield 65%; m.p. 204–207 °C; IR (KBr) *ν*_max_ 3446, 3068, 2945, 1730, 1541, 1456, 1246–1192 cm^−1^; ^1^H NMR (600 MHz, DMSO-d_6_): *δ* 0.81 (3H, s, CH_3_), 0.83 (3H, s, CH_3_), 0.98 (3H, s, CH_3_), 1.02 (3H, s, CH_3_), 1.18 (3H, s, CH_3_), 1.67 (3H, s, CH_3_), 1.78 (3H, s, CH_3_-AZT), 2.03 (3H, s, COCH_3_), 2.55 (1H, m, AZT), 2.67 (1H, m, H-19), 3.65–3.70 (2H, m, AZT), 4.03 (1H, d, *J* = 10.8 Hz, H-28), 4.27 (1H, t, *J* = 4.8 Hz, AZT), 4.38 (1H, m, H-3), 4.55 (1H, d, *J* = 10.8 Hz, H-28), 4.59 (1H, s, H-29), 4.73 (1H, s, H-29), 5.28 (1H, t, *J* = 4.8 Hz, AZT), 5.46 (1H, m, AZT), 6.44 (1H, t, *J* = 6.6 Hz, AZT), 7.82 (1H, s, AZT), 8.32 (1H, s, CH-triazole), 9.01 (1H, s, NH-AZT); ^13^C NMR (150 MHz, DMSO-d_6_): *δ* 12.7, 14.9, 15.9, 16.1, 16.3, 16.9, 18.2, 19.3, 20.8, 21.5, 23.8, 25.3, 27.1, 28.1, 29.4, 29.7, 34.0, 34.6, 37.1, 37.6, 37.7, 37.8, 38.2, 42.8, 46.8, 47.5, 48.7, 49.9, 55.0, 55.6, 60.2, 61.1, 62.7, 79.6, 80.4, 84.6, 110.1, 110.5, 129.3, 136.7, 139.3, 150.9, 160.9, 164.2; 170.6; HRAPCIMS *m/z* (neg): 802.4768 C_45_H_64_N_5_O_8_ (calcd. 802.4755).

##### 3-Acetyl-28-[1-(1-deoxy-β-D-glucopyranosyl)-1*H*-1,2,3-triazol-4-yl]carbonylbetulin (**5f**)

Yield 82%; m.p. 210–212 °C; IR (KBr) *ν*_max_ 3419, 2943, 1732, 1543, 1456, 1246–1191 cm^−1^; ^1^H NMR (600 MHz, DMSO-d_6_): *δ* 0.80 (3H, s, CH_3_), 0.81 (3H, s, CH_3_), 0.83 (3H, s, CH_3_), 0.98 (3H, s, CH_3_), 1.04 (3H, s, CH_3_), 1.68 (3H, s, CH_3_), 1.85 (1H, m, OH), 2.00 (3H, s, COCH_3_), 2.54 (1H, m, H-19), 3.27 (1H, m, OH), 3.39 (1H, m, OH), 3.44 (1H, m, OH), 3.71 (1H, m, CH-sugar), 3.85 (1H, m, CH-sugar), 4.03 (1H, d, *J* = 10.8 Hz, H-28), 4.37 (1H, m, H-3), 4.55 (1H, d, *J* = 10.8 Hz, H-28), 4.59 (1H, s, H-29), 4.63 (1H, m, CH-sugar), 4.73 (1H, s, H-29), 5.20 (1H, d, *J* = 5.4 Hz, CH-sugar), 5.35 (1H, d, *J* = 5.4 Hz, CH-sugar), 5.46 (1H, d, *J* = 5.4 Hz, CH-sugar), 5.61 (1H, d, *J* = 5.4 Hz, CH-sugar), 9.08 (1H, s, CH-triazole); ^13^C NMR (150 MHz, DMSO-d_6_): *δ* 15.0, 16.1, 16.3, 16.9, 18.2, 19.3, 20.8, 21.5, 23.8, 25.2, 27.1, 28.1, 29.4, 29.6, 34.0, 34.6, 37.1, 37.6, 37.8, 38.2, 39.6, 42.8, 46.8, 47.5, 48.7, 50.0, 55.0, 61.2, 62.6, 69.9, 72.4, 77.2, 79.6, 80.6, 88.3, 110.5, 129.1, 139.2, 150.3, 160.9, 170.6; HRAPCIMS *m/z* (neg): 740.4480 C_41_H_62_N_3_O_9_ (calcd. 740.4486).

##### 3-Acetyl-28-(1-ethylacetyl-1*H*-1,2,3-triazol-4-yl)carbonylbetulin (**5g**)

Yield 80%; m.p. 221–224 °C; IR (KBr) *ν*_max_ 2945, 1732, 1544, 1465, 1247–1213 cm^−1^; ^1^H NMR (600 MHz, CDCl_3_): *δ* 0.85 (3H, s, CH_3_), 0.86 (3H, s, CH_3_), 0.88 (3H, s, CH_3_), 0.99 (3H, s, CH_3_), 1.08 (3H, s, CH_3_), 1.34 (3H, t, *J* = 7.2 Hz, CH_3_), 1.68 (3H, s, CH_3_), 2.07 (3H, s, COCH_3_), 2.53 (1H, m, H-19), 4.18 (1H, d, *J* = 10.8 Hz, H-28), 4.32 (2H, q, *J* = 7.2 Hz, OCH_2_), 4.48 (1H, m, H-3), 4.58 (1H, d, *J* = 10.8 Hz, H-28), 4.63 (1H, s, H-29), 4.73 (1H, s, H-29), 5.24 (2H, s, CH_2_), 8.24 (1H, s, CH-triazole); ^13^C NMR (150 MHz, CDCl_3_): *δ* 14.1, 14.8, 16.1, 16.2, 16.5, 18.2, 20.8, 21.3, 23.7, 25.2, 27.1, 27.9, 29.6, 29.8, 34.1, 34.7, 37.1, 37.7, 37.8, 38.4, 40.9, 42.7, 46.7, 47.8, 48.9, 50.3, 51.0, 55.4, 62.8, 63.7, 80.9, 110.0, 128.7, 140.7, 150.1, 160.9, 165.7, 171.1; HRAPCIMS *m/z* (neg): 664.4329 C_39_H_58_N_3_O_6_ (calcd. 664.4326).

##### 3-Acetyl-28-[1-(3-hydroxypropyl)-1*H*-1,2,3-triazol-4-yl]carbonylbetulin (**5h**)

Yield 83%; m.p. 116–119 °C; IR (KBr) *ν*_max_ 3425, 2945, 1732, 1543, 1465, 1246–1199 cm^−1^; ^1^H NMR (600 MHz, DMSO-d_6_): *δ* 0.80 (3H, s, CH_3_), 0.81 (3H, s, CH_3_), 0.82 (3H, s, CH_3_), 0.98 (3H, s, CH_3_), 1.03 (3H, s, CH_3_), 1.09 (2H, m, CH_2_), 1.67 (3H, s, CH_3_), 2.02 (3H, s, COCH_3_), 2.51 (1H, m, H-19), 3.34 (2H, m, CH_2_), 4.01 (1H, d, *J* = 10.8 Hz, H-28), 4.38 (1H, m, H-3), 4.48 (2H, t, *J* = 7.2 Hz, CH_2_), 4.54 (1H, d, *J* = 10.8 Hz, H-28), 4.59 (1H, s, H-29), 4.73 (1H, s, H-29), 8.81 (1H, s, CH-triazole); ^13^C NMR (150 MHz, DMSO-d_6_): *δ* 14.9, 16.1, 16.3, 16.9, 18.2, 19.2, 20.7, 21.5, 24.8, 25.1, 27.1, 28.1, 29.4, 29.6, 33.1, 34.0, 34.6, 37.1, 37.6, 37.8, 38.2, 40.9, 42.8, 46.8, 47.5, 47.6, 48.7, 50.0, 55.0, 57.8, 62.5, 80.4, 110.4, 129.7, 139.0, 150.3, 161.0, 170.6; HRAPCIMS *m/z* (neg): 636.4363 C_38_H_58_N_3_O_5_ (calcd. 636.4376).

##### 2-Amino-3-[4-(3-acetyl-28-betulinylcarbonyl)-1*H*-1,2,3-triazol-1-yl]propanoic acid (**5i**)

Yield 45%; oil; IR (KBr) *ν*_max_ 3444, 2956, 1732, 1602, 1458, 1246–1122 cm^−1^; ^1^H NMR (600 MHz, DMSO-d_6_) *δ*: 0.80 (3H, s, CH_3_), 0.82 (3H, s, CH_3_), 0.86 (3H, s, CH_3_), 0.92 (3H, s, CH_3_), 1.01 (3H, s, CH_3_), 1.06 (1H, t, *J* *=* 7.2 Hz, CH), 1.67 (3H, s, CH_3_), 2.00 (3H, s, COCH_3_), 2.52 (1H, m, H-19), 4.14 (1H, d, *J* = 10.8 Hz, H-28), 4.23 (2H, d, *J* = 7.2 Hz, CH_2_), 4.37 (1H, m, H-3), 4.55 (1H, d, *J* = 10.8 Hz, H-28), 4.58 (1H, s, H-29), 4.73 (1H, s, H-29), 8.62 (1H, s, CH-triazole); ^13^C NMR (150 MHz, DMSO-d_6_): *δ* 14.9, 15.8, 16.1, 16.8, 18.3, 19.2, 19.6, 21.2, 22.9, 23.7, 26.8, 28.8, 30.3, 30.6, 30.8, 34.1, 34.5, 36.8, 37.2, 37.5, 38.5, 39.6, 42.8, 46.2, 47.0, 47.2, 48.6, 50.1, 54.3, 56.5, 67.8, 80.2, 110.0, 129.2, 132.1, 150.1, 161.0, 167.5; HRAPCIMS *m/z* (neg): 665.4269 C_38_H_57_N_4_O_6_ (calcd. 665.4278).

##### 28-(1-Benzyl-1*H*-1,2,3-triazol-4-yl)carbonylbetulone (**6a**)

Yield 81%; m.p. 196–198 °C; IR (KBr) *ν*_max_ 2963, 1738, 1700, 1539, 1465, 1261–1193 cm^−1^; ^1^H NMR (600 MHz, CDCl_3_) *δ*: 0.94 (3H, s, CH_3_), 1.02 (3H, s, CH_3_), 1.05 (3H, s, CH_3_), 1.10 (3H, s, CH_3_), 1.12 (3H, s, CH_3_), 1.69 (3H, s, CH_3_), 2.52 (1H, m, H-19), 4.15 (1H, d, *J* = 10.8 Hz, H-28), 4.57 (1H, d, *J* = 10.8 Hz, H-28), 4.62 (1H, s, H-29), 4.72 (1H, s, H-29), 5.60 (2H, s, CH_2_), 7.31–7.33 (2H, m, H_Ar_), 7.41–7.44 (3H, m, H_Ar_), 7.97 (1H, s, CH-triazole);^13^C NMR (150 MHz, CDCl_3_): *δ* 14.7, 15.8, 15.9, 19.1, 19.6, 21.1, 21.3, 25.2, 25.6, 26.6, 27.1, 29.6, 29.8, 33.5, 34.2, 34.7, 36.9, 37.8, 39.6, 40.9, 42.8, 46.7, 47.4, 47.7, 48.8, 49.7, 54.5, 55.0, 63.5, 68.0, 110.0, 127.1, 128.2, 129.2, 129.3, 133.8, 140.6, 150.0, 161.2, 218.0; HRAPCIMS *m/z* (neg): 624.4171 C_40_H_54_N_3_O_3_ (calcd. 624.4165).

##### 28-[1-(4-Fluorobenzyl)-1*H*-1,2,3-triazol-4-yl]carbonylbetulone (**6b**)

Yield 73%; m.p. 220–223 °C; IR (KBr) *ν*_max_ 3131, 2957, 1742, 1699, 1539, 1456, 1223–1198, 814 cm^−1^; ^1^H NMR (600 MHz, CDCl_3_): *δ* 0.86 (3H, s, CH_3_), 0.91 (3H, s, CH_3_), 0.92 (3H, s, CH_3_), 0.95 (3H, s, CH_3_), 1.02 (3H, s, CH_3_), 1.68 (3H, s, CH_3_), 2.43 (1H, m, H-19), 4.05 (1H, d, *J* = 10.8 Hz, H-28), 4.48 (1H, d, *J* = 10.8 Hz, H-28), 4.53 (1H, s, H-29), 4.63 (1H, s, H-29), 5.48 (2H, s, CH_2_), 7.01–7.04 (2H, m, H_Ar_), 7.23–7.36 (2H, m, H_Ar_), 7.88 (1H, s, CH-triazole); ^13^C NMR (150 MHz, CDCl_3_): *δ* 14.7, 15.8, 15.9, 19.2, 19.6, 21.1, 21.3, 25.2, 26.6, 27.1, 29.6, 29.8, 33.5, 34.2, 34.7, 36.9, 37.8, 39.6, 40.9, 42.8, 46.7, 47.4, 47.7, 48.8, 49.7, 53.7, 55.0, 63.6, 68.1, 110.0, 116.3, 116.5, 126.2, 126.3, 130.2, 140.0, 150.0, 161.1, 218.0; HRAPCIMS *m/z* (neg): 642.4063 C_40_H_53_FN_3_O_3_ (calcd. 642.4071).

##### 28-[1-(4-Cyanobenzyl)-1*H*-1,2,3-triazol-4-yl]carbonylbetulone (**6c**)

Yield 57%; m.p. 211–214 °C; IR (KBr) *ν*_max_ 3127, 2951, 2229, 1734,1705, 1525, 1457, 1243–1147 cm^−1^; ^1^H NMR (600 MHz, CDCl_3_): *δ* 0.87 (3H, s, CH_3_), 0.93 (3H, s, CH_3_), 0.96 (3H, s, CH_3_), 1.02 (3H, s, CH_3_), 1.03 (3H, s, CH_3_), 1.68 (3H, s, CH_3_), 2.44 (1H, m, H-19), 4.07 (1H, d, *J* = 10.8 Hz, H-28), 4.49 (1H, d, *J* = 10.8 Hz, 1H, H-28), 4.54 (1H, s, H-29), 4.63 (1H, s, H-29), 5.59 (2H, s, CH_2_), 7.31 (2H, d, *J* = 8.4 Hz, H_Ar_), 7.63 (2H, d, *J* = 8.4 Hz, H_Ar_), 7.96 (1H, s, CH-triazole); ^13^C NMR (150 MHz, CDCl_3_): *δ* 14.7, 15.8, 15.9, 19.1, 19.6, 21.1, 21.3, 23.7, 25.2, 26.6, 27.1, 29.6, 29.8, 33.5, 34.2, 34.7, 36.9, 37.8, 39.6, 40.9, 42.8, 46.7, 47.4, 47.7, 48.8, 49.7, 53.7, 55.0, 63.7 110.1, 113.3, 117.9, 127.4, 128.5, 133.1, 138.9, 141.0, 149.9, 160.9, 218.1; HRAPCIMS *m/z* (neg): 649.4095 C_41_H_53_N_4_O_3_ (calcd. 649.4118).

##### 28-(1-Phenylthiomethyl-1*H*-1,2,3-triazol-4-yl)carbonylbetulone (**6d**)

Yield 87%; m.p. 188–191 °C; IR (KBr) *ν*_max_ 3132, 2960, 1734, 1705, 1521, 1456, 1241–1196 cm^−1^; ^1^H NMR (600 MHz, CDCl_3_): *δ* 0.96 (3H, s, CH_3_), 1.03 (3H, s, CH_3_), 1.05 (3H, s, CH_3_), 1.09 (3H, s, CH_3_), 1.11 (3H, s, CH_3_), 1.69 (3H, s, CH_3_), 2.53 (1H, m, H-19), 4.15 (1H, d, *J* = 10.8 Hz, H-28), 4.57 (1H, d, *J* = 10.8 Hz, H-28), 4.63 (1H, s, H-29), 4.74 (1H, s, H-29), 5.69 (2H, s, CH_2_), 7.34-7.37 (5H, m, H_Ar_), 8.06 (1H, s, CH-triazole); ^13^C NMR (150 MHz, CDCl_3_): *δ* 14.7, 15.8, 15.9, 19.1, 19.6, 21.1, 21.3, 25.2, 25.6, 26.6, 27.1, 29.6, 29.8, 33.5, 34.2, 34.7, 36.9, 37.8, 39.6, 40.9, 42.8, 46.7, 47.4, 47.7, 48.8, 49.7, 54.3, 55.0, 63.6, 68.0, 110.0, 126.8, 129.1, 129.7, 131.3, 132.4, 150.0, 160.9, 218.1; HRAPCIMS *m/z* (neg): 656.3895 C_40_H_54_N_3_O_3_S (calcd. 656.3886).

##### 28-[1-(3’-Deoxythymidine-5’-yl)-1*H*-1,2,3-triazol-4-yl]carbonylbetulone (**6e**)

Yield 73%; m.p. 199–202 °C; IR (KBr) *ν*_max_ 3447, 3068, 2945, 1729, 1697, 1541, 1458, 1226–1163 cm^−1^; ^1^H NMR (600 MHz, DMSO-d_6_): *δ* 0.88 (3H, s, CH_3_), 0.94 (3H, s, CH_3_), 0.99 (3H, s, CH_3_), 1.02 (3H, s, CH_3_), 1.05 (3H, s, CH_3_), 1.68 (3H, s, CH_3_), 1.74 (3H, s, CH_3_-AZT), 2.51 (1H, m, AZT), 2.67 (1H, m, H-19), 3.65–3.70 (2H, m, AZT), 4.04 (1H, d, *J* = 10.8 Hz, H-28), 4.27 (1H, t, *J* = 4.8 Hz, AZT), 4.55 (1H, d, *J* = 10.8 Hz, H-28), 4.58 (1H, s, H-29), 4.74 (1H, s, H-29), 5.27 (1H, t, *J* = 4.8 Hz, AZT), 5.46 (1H, m, AZT), 6.44 (1H, t, *J* = 6.6 Hz, AZT), 7.82 (1H, s, AZT), 8.32 (1H, s, CH-triazole), 9.02 (1H, s, NH-AZT); ^13^C NMR (150 MHz, DMSO-d_6_): *δ* 12.7, 14.9, 15.8, 15.9, 16.1, 19.3, 19.6, 21.2, 21.3, 25.3, 26.8, 27.1, 29.4, 29.6, 33.4, 34.1, 34.6, 36.8, 37.6, 37.7, 39.3, 39.6, 42.8, 46.8, 47.0, 47.5, 48.6, 49.4, 54.3, 55.6, 60.2, 61.1, 62.7, 79.6, 84.3, 84.7, 110.1, 110.5, 129.3, 136.7, 139.3, 150.3, 150.9, 160.9, 164.2, 217.0; HRAPCIMS *m/z* (neg): 758.4484 C_43_H_60_N_5_O_7_ (calcd. 758.4493).

##### 28-[1-(1-Deoxy-β-D-glucopyranosyl)-1*H*-1,2,3-triazol-4-yl]carbonylbetulone (**6f**)

Yield 74%; m.p. 187–189 °C; IR (KBr) *ν*_max_ 3419, 2939, 1732, 1701, 1541, 1458, 1232–1190 cm^−1^; ^1^H NMR (600 MHz, DMSO-d_6_): *δ* 0.88 (3H, s, CH_3_), 0.94 (3H, s, CH_3_), 0.99 (3H, s, CH_3_), 1.02 (3H, s, CH_3_), 1.07 (3H, s, CH_3_), 1.68 (3H, s, CH_3_), 1.86 (1H, m, OH), 2.56 (1H, m, H-19), 3.27 (1H, m, OH), 3.40 (1H, m, OH), 3.45 (1H, m, OH), 3.71 (1H, m, CH-sugar), 3.85 (1H, m, CH-sugar), 4.04 (1H, d, *J* = 10.8 Hz, H-28), 4.56 (1H, d, *J* = 10.8 Hz, H-28), 4.59 (1H, s, H-29), 4.63 (1H, m, CH-sugar), 4.74 (1H, s, H-29), 5.20 (1H, d, *J* = 5.4 Hz, CH-sugar), 5.34 (1H, d, *J* = 5.4 Hz, CH-sugar), 5.45 (1H, d, *J* = 5.4 Hz, CH-sugar), 5.62 (1H, d, *J* = 5.4 Hz, CH-sugar), 9.08 (1H, s, CH-triazole); ^13^C NMR (150 MHz, DMSO-d_6_): *δ* 14.9, 15.8, 16.1, 19.3, 19.6, 21.2, 21.3, 25.2, 26.8, 27.1, 29.4, 29.6, 33.4, 34.1, 34.6, 36.8, 37.1, 39.3, 42.8, 46.8, 47.0, 47.5, 48.7, 49.4, 54.3, 61.2, 62.6, 69.9, 72.4, 77.2, 79.6, 80.6, 88.3, 110.5, 129.1, 139.2, 150.3, 160.9, 218.0; HRAPCIMS *m/z* (neg): 696.4220 C_39_H_58_N_3_O_8_ (calcd. 696.4224).

##### 28-(1-Ethylacetyl-1*H*-1,2,3-triazol-4-yl)carbonylbetulone (**6g**)

Yield 79%; m.p. 97–99 °C; IR (KBr) *ν*_max_ 3147, 2945, 1755, 1705, 1541, 1458, 1211–1111 cm^−1^; ^1^H NMR (600 MHz, CDCl_3_): *δ* 0.88 (3H, s, CH_3_), 0.93 (3H, s, CH_3_), 0.95 (3H, s, CH_3_), 1.00 (3H, s, CH_3_), 1.03 (3H, s, CH_3_), 1.26 (3H, t, *J* = 7.2 Hz, CH_3_), 1.68 (3H, s, CH_3_), 2.45 (1H, m, H-19), 4.10 (1H, d, *J* = 10.8 Hz, H-28), 4.23 (2H, q, *J* = 7.2 Hz, OCH_2_), 4.51 (1H, d, *J* = 10.8 Hz, H-28), 4.54 (1H, s, H-29), 4.65 (1H, s, H-29), 5.15 (2H, s, CH_2_), 8.16 (1H, s, CH-triazole); ^13^C NMR (150 MHz, CDCl_3_): *δ* 14.2, 14.7, 15.9, 19.6, 21.1, 21.3, 25.2, 26.6, 27.1, 29.6, 29.8, 33.5, 34.2, 34.7, 36.9, 37.8, 39.6, 40.9, 42.8, 46.7, 47.4, 47.7, 48.8, 49.7, 51.0, 55.0, 60.4, 62.8, 63.6, 110.0, 128.7, 140.7, 150.1, 160.9, 165.7, 171.2, 218.1; HRAPCIMS *m/z* (neg): 620.4049 C_37_H_54_N_3_O_5_ (calcd. 620.4063).

##### 28-[1-(3-Hydroxypropyl)-1*H*-1,2,3-triazol-4-yl]carbonylbetulone (**6h**)

Yield 78%; m.p. 197–199 °C; IR (KBr) *ν*_max_ 3404, 2960, 1735, 1703, 1543, 1458, 1261–1223 cm^−1^; ^1^H NMR (600 MHz, DMSO-d_6_): *δ* 0.88 (3H, s, CH_3_), 0.94 (3H, s, CH_3_), 0.99 (3H, s, CH_3_), 1.00 (3H, s, CH_3_), 1.05 (3H, s, CH_3_), 1.10 (2H, m, CH_2_), 1.67 (3H, s, CH_3_), 2.52 (1H, m, H-19), 3.38 (2H, m, CH_2_), 4.02 (1H, d, *J* = 10.8 Hz, H-28), 4.48 (2H, t, *J* = 7.2 Hz, CH_2_), 4.55 (1H, d, *J* = 10.8 Hz, H-28), 4.59 (1H, s, H-29), 4.74 (1H, s, H-29), 8.81 (1H, s, CH-triazole); ^13^C NMR (150 MHz, DMSO-d_6_): *δ* 14.9, 15.8, 16.1, 19.2, 19.5, 21.2, 21.3, 25.2, 26.8, 27.1, 29.4, 29.6, 33.1, 33.4, 34.1, 34.6, 36.8, 37.7, 39.3, 42.8, 46.8, 47.0, 47.5, 47.6, 48.6, 49.3, 54.3, 57.8, 62.5, 79.6, 110.5, 129.7, 139.0, 150.3 161.0, 217.0; HRAPCIMS *m/z* (neg): 592.4131 C_36_H_54_N_3_O_4_ (calcd. 592.4114).

##### 2-Amino-3-[4-(3-acetyl-28-betulonylcarbonyl)-1*H*-1,2,3-triazol-1-yl]propanoic acid (**6i**)

Yield 48%; 163–166 °C; IR (KBr) *ν*_max_ 3479, 2956, 1732, 1705, 1606, 1456, 1280–1223 cm^−1^; ^1^H NMR (600 MHz, DMSO-d_6_) *δ*: 0.85 (3 H s, CH_3_), 0.89 (3H, s, CH_3_), 0.98 (3H, s, CH_3_), 1.02 (3H, s, CH_3_), 1.04 (3H, s, CH_3_), 1.07 (1H, t, *J* *=* 7.2 Hz, CH), 1.67 (3H, s, CH_3_), 2.51 (1H, m, H-19), 4.12 (1H, d, *J* = 10.8 Hz, H-28), 4.21 (2H, d, *J* = 7.2 Hz, CH_2_), 4.56 (1H, d, *J* = 10.8 Hz, H-28), 4.59 (1H, s, H-29), 4.74 (1H, s, H-29), 8.62 (1H, s, CH-triazole); ^13^C NMR (150 MHz, DMSO-d_6_): *δ* 14.3, 15.0, 16.1, 16.3, 16.9, 21.5, 22.9, 23.7, 28.1, 28.8, 30.2, 37.1, 37.8, 38.5, 39.5, 40.8, 42.8, 67.9, 80.4, 110.0, 129.1, 132.1, 132.2, 150.1, 167.5, 217.1; HRAPCIMS *m/z* (neg): 621.4050 C_36_H_53_N_4_O_5_ (calcd. 621.4015).

##### 3-Methyl-3-[4-(28-betulonylcarbonyl)-1*H*-1,2,3-triazol-1-yl]butyric acid (**6j**)

Yield 54%; m.p. 246–249 °C; IR (KBr) *ν*_max_ 3446, 2945, 1732, 1709, 1616, 1456, 1280–1211 cm^−1^; ^1^H NMR (600 MHz, DMSO-d_6_) *δ*: 0.72 (3H, s, CH_3_), 0.87 (3H, s, CH_3_), 0.96 (3H, s, CH_3_), 1.00 (3H, d, *J* = 6.6 Hz, CH_3_), 1.01 (3H, s, CH_3_), 1.04 (3H, s, CH_3_), 1.08 (3H, d, *J* = 6.6 Hz, CH_3_), 1.68 (3H, s, CH_3_), 2.43 (1H, m, CH), 2.53 (1H, m, H-19), 4.03 (1H, d, *J* = 10.8 Hz, H-28), 4.28 (1H, m, CHCOOH), 4.55 (1H, d, *J* = 10.8 Hz, H-28), 4.64 (1H, s, H-29), 4.74 (1H, s, H-29), 8.76 (1H, s, CH-triazole); ^13^C NMR (150 MHz,, DMSO-d_6_) *δ*: 14.3, 14.6, 14.9, 15.8, 16.0, 16.1, 18.9, 19.2, 19.6, 19.9, 21.2, 25.2, 26.8, 27.1, 29.5, 29.7, 30.8, 33.4, 34.1, 36.8, 37.7, 39.3, 40.8, 42.8, 46.8, 47.0, 47.5, 48.6, 49.3, 54.3, 62.6, 79.7, 110.5, 129.4, 138.7, 150.3, 161.3, 217.1; HRAPCIMS *m/z* (neg): 535.3878 C_35_H_51_O_4_ (calcd. 535.3865).

### Biological study

#### Cells

The triterpenes were evaluated for their cytotoxic activity towards three human cancer cell lines: amelanotic melanoma C-32 (ATCC, Rockville, USA), ductal carcinoma T47D (ATCC, Rockville, USA) and glioblastoma SNB-19 (DSMZ, Braunschweig, Germany). The cells were seeded in 96-well plates (Nunc Thermo Fisher Scientific, Waltham, USA) at a density of 5 × 10^3^ cells per well and maintained for 24 h at 37 °C in a humid atmosphere saturated with 5% CO_2_. All cancer cell lines were cultured in DMEM (Lonza, Basel, Switzerland) growth medium containing 10% fetal bovine serum (FBS) (Biological Industries, Cromwell, USA), penicillin (10,000 U/mL) and streptomycin(10 mg/mL) (Lonza, Basel, Switzerland).

#### WST-1 assay

A WST-1 assay (Roche Diagnostics GmbH, Mannheim,Germany) was used for the evaluate of cytotoxicity against the tested human cancer cell lines. The WST-1 assay was carried out after 72 h incubation of the cells with concentrations ranging from 1 to 100 µg/mL of the tested compounds. The WST-1 tetrazolium salt [sodium 2-(4-iodophenyl)-3-(4-nitrophenyl)-5-(2,4-disulfophenyl)-2*H*-tetrazolium] is reduced by mitochondrial dehydrogenases of viable cells to water-soluble formazan. The amount of formazan produced by viable cells was quantified by measuring the absorbance (λ = 450 nm). The anticancer activity of triterpenes were expressed as an IC_50_ in µM (Table 2).

**Table 2:**
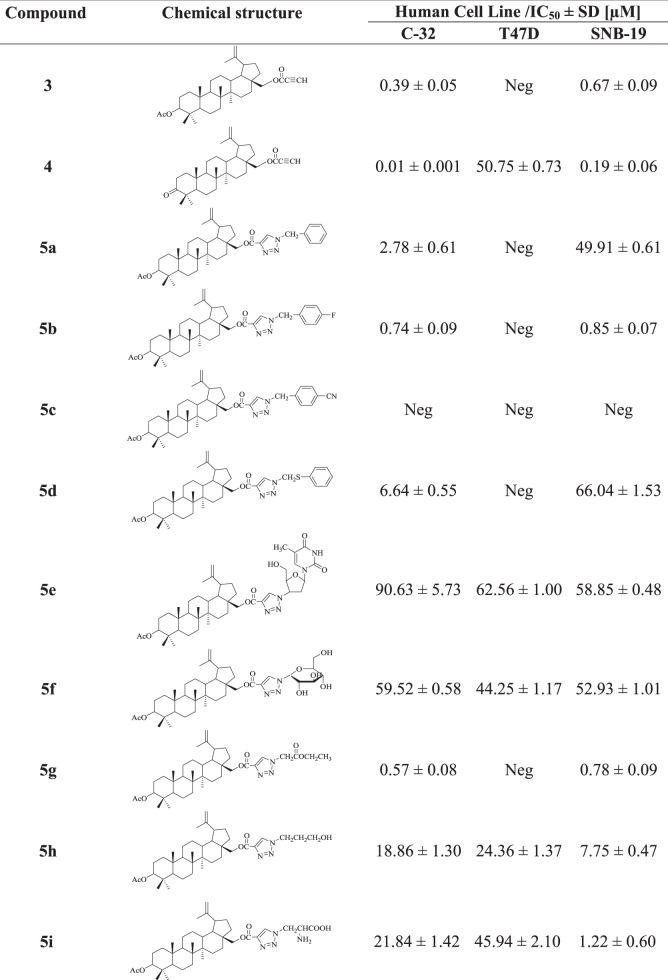

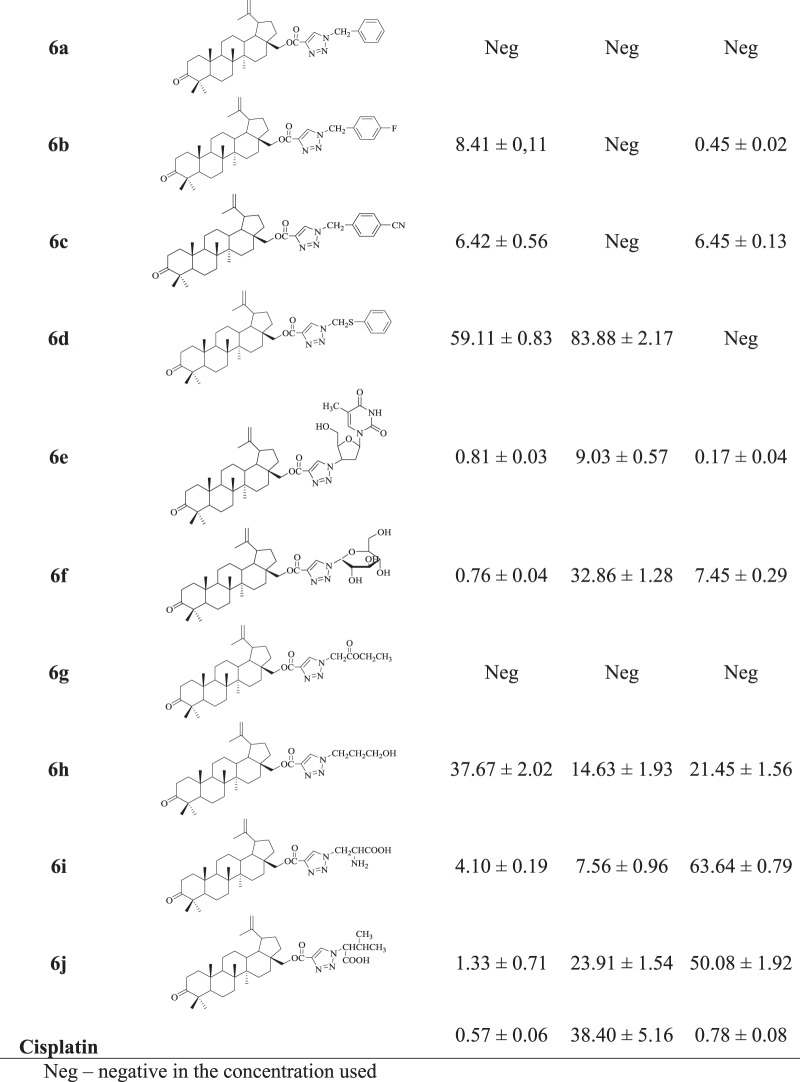
Anticancer activity (IC_50_) of acetylenic esters **3**–**4**, triazoles of triterpenes **5a**-**i** and **6a-j** and cisplatin as a reference compound against the tested human cancer cell lines

### Lipophilicity studies

The theoretical lipophilicity parameters of triazoles **5a**-**i** and **6a-j** were calculated using the commercially available ALOGPS 2.1 software program (Tetko et al. 2005) (Table 3).

**Table 3:** The values of calculated lipophilicity parameters of compound **5a**–**i** and **6a**–**j**

Compound	ALOGPs	AC logP	ALOGP	MLOGP	XLOGP2	XLOGP3
**5a**	7.76	9.09	9.56	7.29	11.43	11.87
**5b**	6.97	7.00	9.13	7.63	10.63	10.95
**5c**	6.91	7.27	8.81	6.78	10.33	10.50
**5d**	7.19	9.60	9.52	7.44	11.03	11.43
**5e**	5.54	4.70	6.46	4.98	7.45	8.30
**5f**	4.54	3.99	5.19	3.99	6.85	6.96
**5g**	6.26	5.78	7.61	6.15	9.03	9.58
**5h**	5.85	5.75	6.87	5.98	8.55	8.80
**5i**	2.66	3.80	6.22	3.03	5.44	5.53
**6a**	6.62	7.13	8.51	6.78	9.26	9.89
**6b**	6.50	7.19	8.72	7.14	9.43	9.99
**6c**	6.58	6.94	8.39	6.39	8.99	9.61
**6d**	6.78	9.28	9.10	7.05	9.69	10.54
**6e**	5.04	4.38	6.04	4.56	6.11	7.41
**6f**	4.16	3.67	4.77	3.56	5.50	6.07
**6g**	5.90	5.45	7.20	5.72	7.68	8.69
**6h**	5.54	5.43	6.45	5.57	7.20	7.91
**6i**	2.37	3.47	5.80	2.59	4.10	4.64
**6j**	6.21	6.00	7.82	5.90	8.12	9.36

## Results and discussion

### Chemistry

**Fig. 1:**
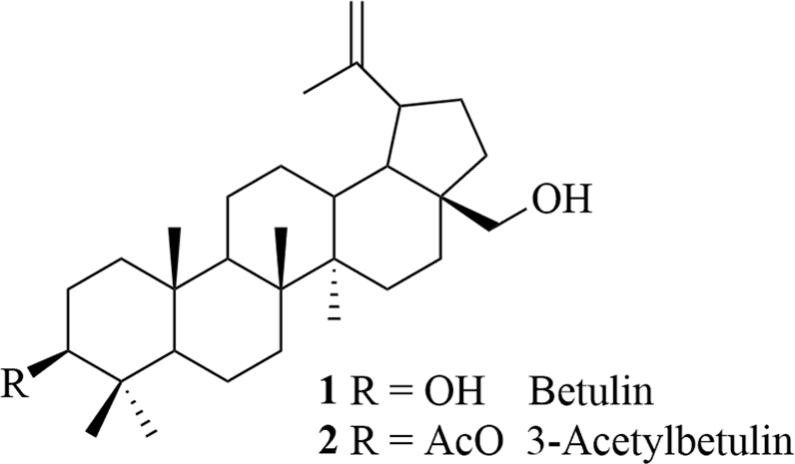
Chemical structure of betulin **1** and 3-acetylbetulin **2**

The synthesis of triazoles was started from betulin **1** and 3-acetylbetulin **2** (Fig. 1). Acetylation of betulin **1** at the C-3 and C-28 positions with acetic anhydride in the presence of 4-dimethylaminopyridine in pyridine gave betulin 3,28-diacetate. A selective hydrolysis of betulin 3,28-diacetate at C-28 position (MeOH/NaOH/THF) afforded 3-acetylobetulin **2** with a quantitative yield (Thibeault et al. 2007; Santos et al. 2010).

**Scheme 1:**
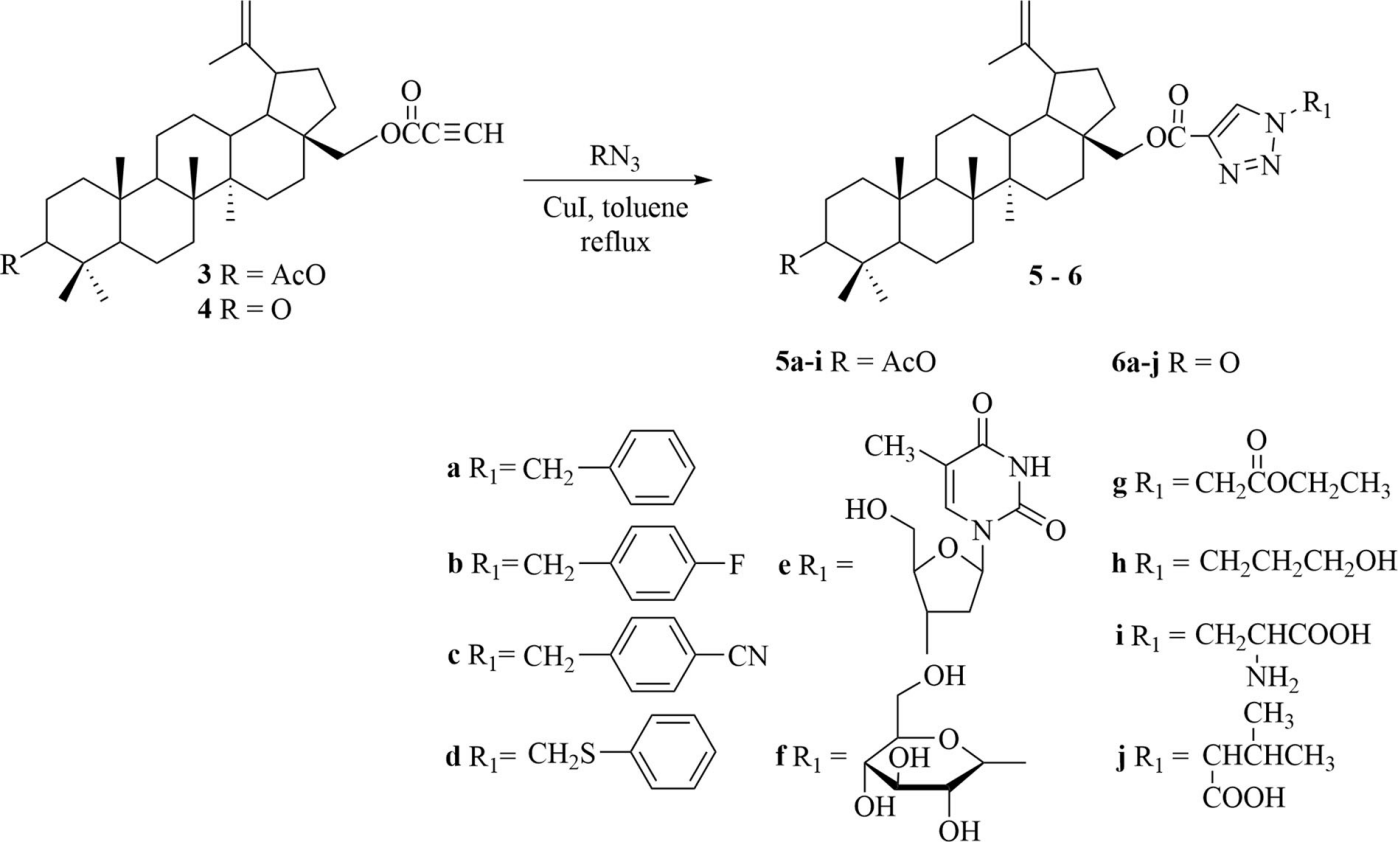
Synthesis of triazole derivatives **5a-i** and **6a**-**j**. *Reagents and conditions*: organic azide (RN_3_), CuI, reflux, 72 h

Subsequently, triterpenes **1**–**2** were used to prepare the propynoylated derivatives **3**–**4** according to our published procedures (Boryczka et al. 2013). The triazoles **5a**-**i** and **6a-j** were obtained by CuAAC reactions of acetylenic esters with various organic azides in toluene with yields in the range of 45–87%. Synthesis of triazoles **5a**-**i** and **6a-j** was depicted in Scheme 1. New compounds were purified by column chromatography on silica gel in CHCl_3_–EtOH or CH_2_Cl_2_–EtOH with various ratios. The chemical characterization of all derivatives was carried out by ^1^H-, ^13^C-NMR, IR spectroscopies, and HRMS spectra.

In the ^1^H NMR spectra of the triazoles **5a**-**d** and **6a**-**d**, singlets of methylene group were observed at *δ* 5.48–5.69, which suggests the presence of a bond between C-4 (aryl group) and N-1 of the triazole ring. The signals in the range of *δ* 7.01–7.72 were assigned to the aromatic protons of the aryl group of derivatives **5a**-**d** and **6a**-**d**. Additionally, for all derivatives **5a**-**i** and **6a-j**, signals at δ 7.96–9.08 were observed, corresponding to triazolyl protons in the 1,4-disubstituted triazole ring.

Analysis of the ^13^C NMR spectra of triazoles **5a**-**i** and **6a-j** showed that the signals of acetyl and carbonyl groups are located at 167.5–171.1 p.p.m. and 217.0–218.1 p.p.m., respectively.

The IR spectra of new triazoles **5a**-**i** and **6a-j** showed characteristic absorption bands at 1527–1616 cm^−1^ and 1456–1458 cm^−1^, which were attributed to the C=N and the N=N stretching vibrations of the triazole ring, respectively.

The HRMS negative mode was applied to identify all new compounds. In the mass spectra of triterpenes **3**, **5a**-**i**, and **6a**-**j** signals based on ions [M−H]^−^ were observed. These signals were corresponding to the calculated values.

### Biological study

The triazole derivatives of 3-acetylbetulin and betulone were evaluated in vitro for their anticancer activity using a WST-1 assay against three human cancer cell lines: amelanotic melanoma C-32, ductal carcinoma T47D and glioblastoma SNB-19. Cisplatin was used as a positive control. The results of the anticancer activity tests of the studied compounds are reported in Table 2 as IC_50_ (µM).

As shown in Table 2, the lowest anticancer activity (IC_50_ 7.56–83.88 µM) of targeted triazoles was observed in the case of the T47D ductal carcinoma cell line. In the tested group of triazoles, derivative **6i** exhibited a highest anticancer activity (IC_50_ 7.56 µM) against the T47D cells, when compared to the positive control.

For triazoles of 3-acetylbetulin **5a**-**i**, the rank order of the anticancer activity towards the C-32 cell line is as follows: **5g** > **5b** > **5a** > **5d** > **5h** > **5i** > **5f** > **5e** > **5c**. The compound 5**g** containing a 1-ethylacetyl moiety in triazole ring had the same anticancer activity against the C-32 cell line as the reference cisplatin (IC_50_ 0.57 µM). Moreover, triazoles **5c**, **6a**, and **6g** had no anticancer activity towards C-32 cell line in the applied concentration range.

According to our studies, compounds **5b**, **5g**, **6b**, and **6e** showed a significant activity against human glioblastoma SNB-19 cell line, with IC_50_ values from 0.17 to 0.85 µM.

The triazole **6e** bearing a 3’-deoxythymidine-5’-yl moiety showed the highest activity in the tested group of compounds against SNB-19 cells, with IC_50_ value of 0.17 µM.

Our studies suggest, that the introduction of acetyl or carbonyl group at the C-3 position of triazole derivatives of triterpenes afforded compounds having a higher anticancer activity against amelanotic melanoma C-32 cell line. Additionally, the compounds **5f** and **6f** containing the 1-deoxy-β-D-glucopyranosyl substituted triazole ring had a better activity than their parent 3-hydroxyl substituted analogs against C-32, T47D, and SNB-19 cell lines (Bębenek et al. 2017).

The lipophilicity is one of the important physicochemical parameters in drug development (Andric and Héberger 2015). A lipophilicity study of the tested triazoles was carried out using the ALOGPS 2.1 software program. The predicted log P values were calculated according to the molecular structures of triazoles **5a**–**i** and **6a**–**j** using six computational methods (ALOGPs, AC logP, ALOGP, MLOGP, XLOGP2, and XLOGP3). Considering two triazoles of betulone **6d** and **6e**, it was observed that their cytotoxic activity increased with the decreasing value of theoretical log *P*.

## Conclusion

In conclusion, on the basis of the CuAAC reaction, a series of new derivatives of 3-acetylbetulin and betulone bearing 1,2,3-triazole moiety has been synthesized. The anticancer activity of the triazoles and cisplatin was tested against the C-32, T47D and SNB-19 cancer cell lines using the WST-1 assay. The triazole **6e** with 3’-deoxythymidine-5’-yl substituent proved to be a potent cytotoxic agent with IC_50_ value of 0.17 µM in the case of the human glioblastoma SNB-19 cell line. Morever, the triazole **6e** can be considered as a promising candidate for anticancer therapy.
